# Intraosseous infusion of acyclovir in a neonate

**DOI:** 10.1186/s13052-022-01353-z

**Published:** 2022-09-06

**Authors:** Saverio De Marca, Matteo Calafatti, Luciana Romaniello, Simona Pesce, Rosa Lapolla, Camilla Gizzi

**Affiliations:** 1 Department of Neonatology and NICU, “San Carlo” Hospital, Potenza, Italy; 2 Sapienza University of Rome, Piazzale Aldo Moro 5, 00185 Rome, Italy; 3 Department of Neonatology and NICU, “Sant’Eugenio” Hospital - ASL RM2, Rome, Italy

**Keywords:** Case report, Meningitis, Intraosseous, Infusion, Acyclovir, Neonate

## Abstract

Intraosseous (IO) access offers a fast and reliable route for administration of fluids and drugs when intravenous (IV) accesses like umbilical, peripheral, or peripherally inserted central lines fail in critically ill neonates. Several medications can be successfully administered via the IO route, however only limited information is available regarding IO administration of antiviral agents.

We present the case of a 2-week-old neonate, admitted to the Neonatal Intensive Care Unit (NICU) due to suspected meningitis, who received acyclovir through IO infusion after the venous access was lost and a new one could not be established. No complications were reported within 12 months of follow up.

This report highlights the feasibility of IO acyclovir infusion when IV accesses fail in a critically ill neonate.

Dear Editor,

We present the first-ever report of intraosseous (IO) infusion of acyclovir in a critically ill neonate for the treatment of suspected meningitis following the loss of peripheral intravenous (IV) access.

Prompt aggressive antimicrobial intervention is lifesaving in neonates with suspected meningitis. Because distinguishing viral from bacterial meningitis is difficult in the early clinical course, a combination of agents is often necessary to provide coverage for both types of infection. For critically ill infants, in whom a venous access is difficult or impossible to establish, IO infusion provides a readily available route of drug administration to maintain drug coverage timely and effectively. Several medications can be successfully administered via the IO route [1, 2], however only limited information is available regarding IO administration of antiviral agents. To the best of our knowledge, there have been no previous reports on IO infusion of acyclovir in neonates.

A 12 days old, 3290 g female term infant was admitted to our Neonatal Intensive Care Unit (NICU) with fever and lethargy. She was born by vaginal delivery after an uneventful pregnancy. Maternal rectovaginal swabs were positive for Group B Streptococci (GBS) and a complete ampicillin course (2 g followed by 1 g each 4 hours, for a total of 3 doses) was administered before delivery. Her gestational age and birth weight were 40 weeks and 3095 g, respectively. Clinical history from birth to admission was unremarkable. During the last 12 hours prior to admission, the infant showed feeding problems, lethargy and fever. Upon admission to the NICU, her temperature measured 38.8 °C. A physical examination revealed a lethargic, hyporeactive and poor perfused infant with a slightly bulging bregmatic fontanelle. Initial blood tests revealed increased C-reactive protein (CRP) (21 mg/L; n.v. 0-8 mg/L) and haemoglobin, total leukocyte count, serum electrolytes, renal and liver function tests within normal limits. Suspecting a Central Nervous System (CNS) infection, a lumbar puncture was taken. The spinal tap resulted in a clear cerebrospinal fluid (CSF) sample with 1916 leucocytes/mm^3^ (55% polymorphs and 45% lymphocytes). Glucose level in CSF was reduced (20 mg/dL) while proteins were increased (204 mg/dL). Blood and CSF specimens were cultured for common bacterial pathogens of the CNS. Blood and liquor specimens were also sent to an external laboratory to be examined by PCR for common viral pathogens of the CNS (including Herpes Simplex Virus 1 and 2 (HSV1 and HSV2). After performing blood and CSF analyses, an IV access was obtained and infusion of empiric antibiotic (ampicillin, netilmicin and ceftazidime) and antiviral therapy was started, as per protocol in use in our unit. Acyclovir (20 mg/kg every 8 hours) was administered in 20 mL of 0.9% sodium chloride as hourly IV infusions. The infant also received fluid therapy. Unfortunately, 20 hours later, her venous access was lost and a new one could not be obtained due to vessel fragility. In order to timely and effectively administer the therapy, an IO line was then placed in the patient’s proximal right tibia by means of an orthopaedic drill (ARROW EZ-IO, Pediatric Intraosseous Vascular Access System - Teleflex, Morrisville, USA). The IO access was kept in place for 8 hours, until a femoral vein cannulation was established. The infant received a single dose of ampicillin, ceftazidime and acyclovir through the IO access. While the IO access was in place, the infant’s extremity was regularly checked for swelling, extravasation or altered circulation. Transcranial Ultrasound (US) through the anterior fontanelle performed 30 hours after admission detected echogenic widening of brain sulci and interhemispheric fissure associated with meningeal thickening and mild ventricular enlargement. CSF culture results revealed GBS meningitis. She was managed accordingly and discharged 25 days later. A Magnetic Resonance Imaging (MRI) performed on day 12 of her hospital stay showed mild ventricular enlargement as well as left frontal and temporal, and bilateral interhemispheric, meningeal enhancement after IV contrast. One month later, control MRI indicated a complete resolution of the inflammatory process. The infant has been regularly followed up until the age of 12 months.

Acyclovir is a guanine analogue antiviral drug. The IV solution is highly alkaline (pH 11, osmolality 278 mOsm/kg), therefore diluting the concentration of acyclovir before infusion is commonly indicated. Despite dilution, dermatological adverse effects due to solution extravasation, such as erythema, inflammation, phlebitis at the injection site, and bullous eruptions have been described [1]. For the same reasons, acyclovir could potentially damage the intraosseous space during IO infusion. The potential for acyclovir-specific adverse effects and the risks intrinsic to the establishment of an IO access limit the employment of their combination in conventional clinical practice. However, in emergent/urgent conditions or in medically necessary cases, the benefits tend to outweigh the risks. No complications were noticed in our patient as a result of the procedure. The patient has been regularly checked during her hospital stay and followed-up until 12 months of life. Her clinical course was unremarkable.

The IO access was first used in 1922, and first described in pediatric patients in 1947 [2]. Despite the relatively little neonatal-specific literature on IO access, the reassuring body of knowledge coming from the scientific literature about the safety of the procedure, along with the availability of dedicated and easy-to-use devices, contributed to its widespread diffusion. For this reason, in recent years IO access in neonates and infants has increasingly gained consensus. The IO access can be also considered for preterm newborns [3].

The IO route is indicated any time a vascular access is difficult to obtain in emergent/urgent conditions or in medically necessary cases (i.e., sedation for procedures, antibiotic administration, analgesia, blood sampling, etc). In neonatal units, umbilical venous catheterization (UVC) is a reliable method of delivering emergency drugs and fluids. However once the umbilical vessels have closed and the cord stump has dried and shriveled, UVC access becomes almost impossible to establish. In these instances, as well as when attempts at securing other IV accesses have failed, IO access warrants exploration as an alternative. Indeed, the 2021 European Resuscitation Council Guidelines on Paediatric Life Support recommend the IO access for infants and children, as the primary rescue alternative [4]. Similarly, two recent systematic literature reviews concluded that IO access should be available on neonatal units and considered for early use in neonates where other access routes have failed [3, 5].

Several devices are available for IO access [3], including IO needles and semi-automatic drills. Anatomical investigation in stillborns reported that the semi-automatic drill, although easier to use, demonstrated lower success rates than the IO needles in establishing IO access. A subsequent randomized simulation study on a on a neonatal bone model confirmed these results [6]. The principle of IO access is to insert a needle into the medullary cavity of a long bone. The suggested neonatal IO insertion site is the proximal tibia (antero-medial surface of the tibia 10 mm below the tibial tuberosity), as there is an optimal (minimal) cortical thickness-to-medullary cavity ratio, fewer critical structures are at risk, and neither is the blood flow to the lower limbs compromised. However, in infants, the distal tibia may be also selected. More recently, the superolateral aspect of the humerus and distal femoral end have been considered as an alternative IO site in newborns [7]. In inserting the IO needle, care must be taken in infants and young children to avoid the epiphyseal plates. The IO needle may remain in place for up to 24 hours [3] and its position may be confirmed by any of the following: aspiration of bone marrow, checking that the device is free-standing in the bone, or infusing a small volume of fluid under direct vision and monitoring for extravasation. Once the access is established, the use of the same drug and fluid doses is suggested whether administered by IO or IV route.

Complication rates (13-31%) amongst the infants in published case reports and case series are higher than those quoted in studies for older patients [3]. Whilst this may be reflective of the more severe cases lending themselves to case reports, complication and failure rates of IO device placement are higher in younger patients*.* A review of tibial IO insertion in pediatric emergency care based on postmortem Computed Tomography imaging reported a 53% failure rate (non-medullary placement) in infants 6 months of age or younger [8]. This may be partly explained by the smaller size of the medullary cavity***.*** The complications associated with IO devices include mispositioned needles, displaced needles, extravasation, infection (local infection or osteomyelitis), fracture, compartment syndrome, and more rarely limb ischemia requiring amputation and fat or air emboli. Among these, the most common include extravasation of fluids, compartment syndrome, and osteomyelitis [3]. For these reasons, the IO insertion site should be monitored frequently for any signs of extravasation or altered circulation. Overall, the risk of such complications may only be acceptable in emergencies which are potentially life-threatening [3] or otherwise at risk of severe complications, when there are no other forms of venous access available. Literature data shows that uncomplicated IO route has no long-term negative effects on the bone, growth plates and marrow elements in the pediatric population [9]. After IO needle removal, fibrin and clotting prevent extravasation from the bone hole. Subsequent sealing of the bone occurs in about 48 hours. Complete healing ranges from days to weeks [10].

In conclusion, to the best of our knowledge, the literature regarding the safety and the effectiveness of IO acyclovir in neonates is absent. Our case is limited to a single 20 mg/kg dose of IO acyclovir administration with no adverse effects reported throughout a 12-months follow up. Further clinical studies are needed to confirm the safety and efficacy of acyclovir administration through this route, in order to highlight the risks and benefits for infants when IO access is the only available option.

## Data Availability

The data used and/or analyzed during the current study are available from the corresponding author on reasonable request.

## Abbreviations

IO: Intraosseous
IV: Intravenous
GBS: Group B Streptococci
NICU: Neonatal Intensive Care Unit
CRP: C-Reactive Protein
CNS: Central Nervous System
CSF: Cerebrospinal fluid
HSV1 and HSV2: Herpes Simplex Virus 1 and 2
US: Ultrasound
MRI: Magnetic Resonance Imaging
UVC: Umbilical venous catheterization

## References

[1] Neocleous C, Andonopoulou E, Adramerina A (2017). Tissue necrosis following extravasation of acyclovir in an adolescent: a case report. Acta Med Acad.

[2] Heinild S, Tyge S, Tudvad F (1947). Bone marrow infusion in childhood. J Pediatr.

[3] Scrivens A, Reynolds PR, Emery FE (2019). Use of intraosseous needles in neonates: a systematic review. Neonatology..

[4] Van de Voorde P, Turner NM, Djakow J (2021). European resuscitation council guidelines 2021: Paediatric life support. Resuscitation..

[5] Wagner M, Olischar M, O’Reilly M (2018). Review of routes to administer medication during prolonged neonatal resuscitation. Pediatr Crit Care Med.

[6] Keller A, Boukai A, Feldman O (2022). Comparison of three intraosseous access devices for resuscitation of term neonates: a randomised simulation study. Arch Dis Child Fetal Neonatal.

[7] Eifinger F, Scaal M, Wehrle L (2021). Finding alternative sites for intraosseous infusions in newborns. Resuscitation..

[8] Carlson JN, Gannon E, Mann NC (2015). Pediatric out-of-hospital critical procedures in the United States. Pediatr Crit Care Med.

[9] Fiser DH (1990). Intraosseous infusion. *N Eng*. J Med.

[10] Ginsberg-Peltz J (2016). Time to bone healing after intraosseous placement in children is ill defined. Pediatr Emerg Care.

